# Fatal spontaneous rectus sheath hematoma in a patient with cirrhosis

**DOI:** 10.4103/0974-2700.66550

**Published:** 2010

**Authors:** Danielle M McCarthy, Shashi Bellam

**Affiliations:** Department of Emergency Medicine, Northwestern University Feinberg School of Medicine, Northwestern Memorial Hospital, Chicago, IL, USA; 1 University of Chicago Pritzker School of Medicine, NorthShore University Health System, Evanston, IL, USA

**Keywords:** Abdominal wall hematoma, cirrhosis, rectus sheath hematoma

## Abstract

Rectus sheath hematoma (RSH) is an uncommon and often misdiagnosed condition. This well-described entity is typically self-limited. In rare cases, the condition may be fatal. We report a case of a patient with cirrhosis who died of progressive RSH and its subsequent complications.

## INTRODUCTION

Rectus sheath hematoma (RSH) is an uncommon condition with a typically benign clinical course. We report the case of a patient with cirrhosis, who developed shock and hepatorenal syndrome as a result of RSH.

## CASE REPORT

A 56-year-old female with a past medical history of cryptogenic cirrhosis presented to the emergency department from her primary doctor’s office with a complaint of abdominal pain. She had symptoms of nasal congestion and “violent” coughing for several days. The patient was treated with moxifloxacin and antitussives and then she developed abdominal pain. The abdominal pain increased with both movement and coughing. She noted some abdominal wall bruising on the day of presentation.

On review of outpatient records, her cirrhosis was fairly well compensated. She had one prior hospitalization for hyperbilirubinemia that had resolved. Lab tests done 1 month prior to presentation revealed thrombocytopenia with a platelet count of 90,000/ml, but were otherwise normal. A computed tomography (CT) scan of the abdomen 1 month prior to presentation revealed cirrhosis with numerous varices, but no ascites. The patient had no history of abdominal surgeries and had a Body Mass Index (BMI) of 23. The patient was tachycardic with a heart rate of 119 on arrival, but was normotensive with a BP of 126/56. The physical exam revealed a left-sided abdominal wall hematoma, and additionally had an area of ecchymosis tracking down the left abdominal wall to the labia. Given the patient’s history of thrombocytopenia, tachycardia and the extent of the ecchymosis, a CT scan of the abdomen, labs and surgical consultation were obtained from the Emergency Department (ED).

The CT scan of the abdomen revealed a large RSH. The first set of labs revealed a hemoglobin level of 10.6 mg/dl and platelet count of 146,000/ml. The INR was within normal limits at 1.2, Prothrombin time (PT) w as 11.7, Partial Thromboplastin Time (PTT) was 26, and the bilirubin was elevated to 8.7 mg/dl, but other liver function tests were normal. The surgical consultants recommended conservative management with pain control, serial hemoglobin levels and observation. The patient’s tachycardia improved in the ED and she remained hemodynamically stable, so she was admitted to the medical floor for pain control and observation. On day 2 of hospitalization, the patient became hypotensive and her systolic blood pressure dropped to the 60s and she developed an associated tachycardia with a heart rate of up to 150. The hemoglobin dropped several grams on repeat blood work and she required transfusion. At that time she was transferred to the intensive care unit.

**Figure 1:**
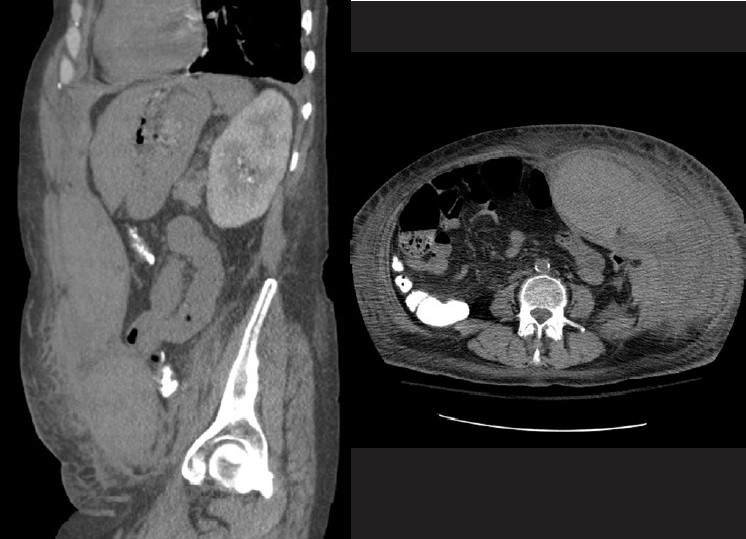
CT Scan images of abdominal wall hematoma (H)

**Figure 2:**
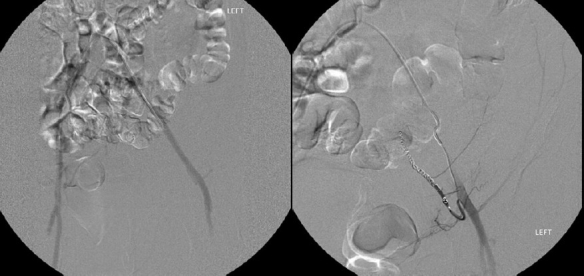
Angiography images of the left inferior epigastric artery

A repeat CT scan of the abdomen revealed a worsening RSH involving the transverse abdominis, internal oblique, and external oblique muscles, and extended from the pelvis to the mid left hepatic lobe, spanning 19.1 × 6.4 × 5.2 cm [Figure 1]. The surgical consultants recommended interventional radiology (IR) evaluation. Given the extent of the hematoma and the fact that the source of bleeding was not identified on CT, it was felt that IR would be best able to identify and treat the bleeding vessel(s). Selective angiography revealed multiple areas of contrast extravasation from the left inferior epigastric artery that was subsequently embolized [Figure 2].

Post-procedure, the patient demonstrated an appropriate response to transfusion with improved hemodynamics. Approximately 36 hours following the embolization, the patient again became tachycardic and hypotensive and a subsequent CT revealed increased size of the hematoma, possible hemopericardium, and possible intraperitoneal blood. On day 5 at the hospital, repeat angiography demonstrated contrast extravasation from the distal inferior mesenteric artery as well as the superior epigastric artery that were subsequently embolized.

Over the following days, the hemoglobin continued to slowly decline and liver function worsened requiring frequent transfusions of fresh frozen plasma (FFP) and platelets. She developed marked hyperbilirubinemia which was suspected to be related to the re-absorbing hematoma. The patient developed hepatic encephalopathy and hypoxemic respiratory failure requiring mechanical ventilation. She went on to develop oliguric renal failure that was consistent with the hepatorenal syndrome. Bladder pressures were monitored and an abdominal compartment syndrome was ruled out.

The Critical Care team consulted the following services for assistance in managing this complicated patient: IR, Surgery, Gastroenterology/Hepatology, Hematology, Nephrology, and Infectious Disease. After two embolization procedures, further embolization via IR was not considered a viable option because of the high risk for ischemia of the rectus abdominis and abdominal wall. The surgical consultants felt that the bleeding had been arterial and not variceal, based on the arteriograms. They found the lack of varices in the rectus sheath and also that the size of the hematoma would easily compress variceal bleeding. Even with portal hypertension, the pressure of variceal bleeding would not exceed 20–30 mm Hg. Based on this reasoning, they did not believe that transjugular intrahepatic portosystemic shunt (TIPS) was indicated. Gastroenterology/Hepatology team agreed with this assessment. Other surgical options were explored, including evacuation of the hematoma, as well as liver transplantation. Ultimately, the patient’s hemodynamics had stabilized on day 9 at the hospital, and as a result surgery was not pursued.

Evaluations for dysfibrinogenemia and Disseminated Intravascular Coagulation (DIC) were negative. Throughout hospitalization, she required transfusion of 19 U packed red blood cells, 10 U platelets, 17 U FFP, two doses of Desmopressin (DDAVP) and cryoprecipitate. Evaluation for infection was also negative. The patient had bilateral infiltrates on chest radiography; however, this was thought to be secondary to volume overload or transfusion related acute lung injury. She was covered empirically for sepsis with broad-spectrum antibiotics. Her hemodynamic monitoring with Swan-Ganz catheterization showed adequate cardiac output and mixed venous saturation monitoring; she never demonstrated signs of distributive shock. Blood and urine cultures obtained throughout the hospitalization were negative.

On day 9 of hospitalization, the patient had become hemodynamically stable and, therefore, the bleeding was believed to have stopped; however, at that point the patient had developed multisystem organ failure. Ultimately, the patient’s family decided to withdraw medical support rather than initiate dialysis and/or pursue liver transplantation. With the understanding that due to her depressed mental status and poor oxygenation the patient was dependent upon mechanical ventilatory support, the family elected to focus on comfort care. The patient was extubated and she expired shortly afterward on day 12 of hospitalization.

## DISCUSSION

RSH is an uncommon condition caused by hemorrhage into the rectus sheath. This diagnosis was first described during the period of Galen and Hippocrates.[1,2] While typically being a self-limited condition, RSH can be a life-threatening condition depending on the size of the bleed and co-morbid conditions as demonstrated by our case. This can be an elusive diagnosis that may mimic many other forms of intra-abdominal pathology.

In our patient, a muscular tear of the rectus related to coughing likely caused the RSH and hemorrhage from the epigastric arteries, as evidenced by the angiography. Several case reports and small case series have looked at the factors that precipitate RSH and have found that it is most often related to minor abdominal trauma, exertional abdominal wall straining (such as coughing), and iatrogenic causes (such as intra-abdominal injections or laparoscopic surgery).[3,4] Anticoagulation therapy has also been implicated as a risk factor for RSH.[5,6] It is unknown if coagulopathies caused by liver disease increase bleeding in RSH.

The most important step in correctly diagnosing RSH is to entertain the diagnosis. In Teske’s series of 100 patients, only 17 were accurately diagnosed prior to surgery; the disease was misdiagnosed in up to 90% of cases.[1] The history described in the case report above is the “typical” initial presentation. The physical examination may reveal abdominal wall ecchymosis. Rarely is the ecchymosis as extensive as in our presentation, tracking down to the groin. Our patient did not have Fothergill’s sign, fixation of the tenderness in the abdominal wall elicited by contraction of the abdominal musculature. The Fothergill sign is considered pathognomonic for abdominal wall masses because fixation with muscle contraction indicates that the mass is in the abdominal wall, rather than being intra-abdominal.

Familiarity with the anatomy of the rectus sheath is important to be considered in our case because several questions regarding the mechanism and location of bleeding remain unanswered in our case. The rectus abdominis muscle extends from the fifth rib superiorly to the pubic bone and is enclosed within a muscular aponeurosis. The arcuate line functionally separates the rectus sheath into a superior and an inferior portion and is located approximately 5 cm below the umbilicus. Below the level of the arcuate line, the aponeuroses of the rectus muscle remain intact anteriorly; however, posteriorly, the only separation between the muscle and abdominal viscera is the weak transversalis fascia and peritoneum. We know that the blood supply to the muscle is from the superior and inferior epigastric arteries and these same arteries were embolized by IR as described above. It remains unknown why this patient repeatedly bled after embolization with normal coagulation factors. Given the patient’s history of portal hypertension, the possibility for a venous source of bleeding in our case was investigated; however, angiography never demonstrated a venous source. The patient may have had an enlarged splanchnic blood pool and congestion, as is often seen in cirrhotic patients; however, it also remains unclear if this contributed to her re-bleeding and shock.

Treatment of uncomplicated RSH is typically conservative with rest, analgesics, ice, compression of hematoma, and treatment of underlying predisposing conditions. In patients who are hemodynamically unstable or with expanding hematomas, intervention is recommended. In the past 5 years, the literature has described the successful use of selective embolization to control active bleeding.[7] Surgery may be considered for patients who are hemodynamically unstable. This procedure is often a last resort and can be limited by the ability to visualize and ligate the bleeding vessels.

RSH is typically well tolerated by patients with conservative treatment. Fatality from RSH is uncommon, but has been reported in the literature.[5,8,9] The overall reported mortality rate in both surgical and conservatively managed patients is estimated to be 4% with increased mortality in patients on anticoagulation and those requiring surgical intervention.[8]

Several other patients with cirrhosis and RSH have been reported in the literature.[4,9,10] The physiologic changes that accompany cirrhosis and ensuing coagulopathy suggest that this subgroup of patients might experience excess morbidity from RSH; however, there are no sufficient published data to quantify this risk for morbidity and mortality.

## CONCLUSION

The above case reveals that RSH, which typically follows a benign clinical course, may be fatal in the cirrhotic patient. The effect of coagulopathy and portal hypertension on the risk and clinical course of RSH is unclear. Similarly, the possible roles of TIPS and liver transplantation in treatment need to be further explored.^10^ RSH is a potentially devastating diagnosis in the cirrhotic patient and further investigation into innovative treatments for this subset of patients is needed.
